# A bibliometric analysis of environmental behavior (1974–2024): evolution of research hotspots and trends

**DOI:** 10.3389/fpsyg.2025.1662745

**Published:** 2025-12-05

**Authors:** Shanshan Wang, Gangtian Liu, Jun Ma, Hongyu Li, Wenao Li, Huijie Bu, Peng Chen, Peng Song, Qiaoxue Chen

**Affiliations:** 1 School of Art and Design, Henan University of Science and Technology, Luoyang, China; 2 College of Agriculture, Henan University of Science and Technology, Luoyang, China; 3 College of Liberal Arts, Nankai University, Tianjin, China

**Keywords:** environmental behavior, pro-environmental behavior, green human resource management, bibliometric analysis, research hotspots

## Abstract

In recent years, environmental behavior has emerged as a crucial research direction for achieving sustainable development. Promoting environmental awareness and related practices can effectively reduce pollution, protect ecosystems, and enhance human well-being. A systematic understanding of research hotspots and evolutionary trends in this field holds significant theoretical and practical value. This study employed bibliometric methods and utilized ScientoPy and VOSviewer analysis tools to conduct a systematic and multi-dimensional analysis of 6,524 articles on environmental behavior published in the Web of Science and Scopus databases from 1974 to 2024. The findings show that the volume of literature on environmental behavior has grown rapidly, confirming its interdisciplinary and global nature. Diachronic clustering analysis identified three distinct evolutionary stages. China ranked first and the United States second in terms of the total number of publications. Current emerging hotspots with the fastest growth include “green human resource management” and “environmental awareness.” Meanwhile, “pro-environmental behavior,” “sustainability,” “climate change,” and “place attachment” remain consistently high-potential research themes. This study comprehensively reveals the historical evolution and shifting hotspots in environmental behavior research over the past 50 years. The findings provide strong evidence and clear directions for scholars, policymakers, and practitioners.

## 1 Introduction

Environmental behavior refers to the patterns of behavior exhibited by individuals or groups in both the natural and social environments. These behaviors interact with and are mutually influenced by the environment, affecting it while also being affected by it. Environmental behavior includes both “pro-environmental behaviors” or “environmentally friendly behaviors,” which are beneficial to the environment (such as conserving resources and participating in environmental protection activities), and “non-environmentally friendly behaviors,” which are harmful to the environment (such as over-consumption and indiscriminate waste disposal). With the emergence of issues like climate change, biodiversity loss, plastic pollution, water scarcity, air pollution, and land degradation and desertification, the world has continually faced serious environmental challenges (Asah et al., 2014; Jagers et al., 2014; Agoston et al., 2022; Abdelwahed et al., 2022). Environmental behavior is a crucial aspect of behavioral management, closely linked to citizens’ actions and intentions, and thus holds significant importance in addressing environmental problems. The pivotal role of environmental behavior is increasingly evident in responding to these global challenges. Understanding the driving forces, sociocultural contexts, and dynamic changes underpinning environmental behavior is essential. This knowledge contributes to designing effective environmental education strategies, formulating precise environmental policies, and promoting large-scale, sustainable behavioral change (Price et al., 2014; Fujii, 2006). This field examines human-environment interactions and draws from disciplines like environmental psychology, ecology, and sociology. With the emergence of the Hygienist Movement, in Europe from the late 19th century to the early 20th century, psychologists and sociologists began exploring environmental influences on people (Weisman, 1983; Hsia, 1988; Pol, 2006; Pol, 2024). For instance, Hellpach introduced the idea of “geopsychology,” highlighting nature’s effects on mental states, and later coined the term “environmental psychology” (Hellpach, 1924; Kaminski, 1976). The 1960s marked the rise of this discipline, with Barker proposing that certain settings trigger predictable behaviors (Barker, 1968). The 1970s environmental movement spurred studies on attitudes toward sustainability, including practices like energy conservation and recycling, which used theories of planned behavior to explain eco-friendly choices. From the 1990s to the 2000s, global concerns drove practical research for urban planning and policy, examining roles of education, incentives, technology, and cultural variations. Since 2010, climate shifts and digital tools have advanced the field, focusing on subtle environmental tweaks, mental health effects, and group efforts to handle crises. Although there have been an increasing number of valuable studies accumulated in various databases (Bharani et al., 2024; Bimonte et al., 2022; Breunig and Russell, 2020), researchers have already explored environmental behavior from multiple dimensions such as “environmental awareness,” “environmental behavior,” “green theory,” “pro-environmental behavior,” and “environmental education” (Abd Rauf et al., 2021; Zhang et al., 2024; Yin et al., 2024). However, bibliometric studies comprehensively evaluating this field remain scarce and limited, often constrained by single data sources, limited time spans, and a lack of dynamic tracking of thematic evolution. This has resulted in an incomplete understanding of the field’s developmental trajectory, knowledge structure, research hotspots, and emerging trends. To address these gaps and provide guidance for future research, a systematic review of environmental behavior studies is imperative. This study quantitatively reviews and sorts out the research on environmental behavior literature over the past 50 years through bibliometric quantification, quantitative analysis, and scientific mapping visualization analysis. By constructing a scientific and reasonable analytical framework, it quantitatively assesses the research achievements in the field of environmental behavior, and at the same time, uses scientific mapping visualization technology to visually present the knowledge structure and evolution of research hotspots in this field (D'oca et al., 2017; Cheung and Hui, 2018; Wang and Li, 2022; Yu S. et al., 2023; van de Ven et al., 2018; Gould et al., 2016; Park et al., 2020). The aim is to reveal the development trajectory of environmental behavior research, identify key research themes and their dynamic changes, and provide strong data support and theoretical references for subsequent research.

Bibliometrics is a widely employed methodology for the quantitative description, evaluation and monitoring of data. It can provide reasonable and objective results while minimizing the subjective biases of researchers (Bell-James, 2023). In recent years, this approach has become increasingly popular. Bibliometric analysis primarily includes two aspects: quantitative analysis and scientific mapping visualization analysis. Quantitative analysis involves the statistical examination of document counts, author contributions, institutional affiliations, journal distributions, and country-specific outputs, thereby revealing the developmental trajectory, prominent research hotspots, and emerging trends within a specific research domain (Lyu et al., 2023; Ninkov et al., 2022; Yu Y. et al., 2023). Complementarily, scientific mapping visualization utilizes visual representations such as network maps to intuitively display crucial information pertaining to knowledge structures, interdisciplinary connections, and research frontiers. This visual approach enables researchers to rapidly understand the overall context and key components of a research field, effectively revealing its underlying knowledge architecture and evolutionary process (Quevedo et al., 2023; Costa and Macreadie, 2022; Jiang et al., 2022).

In this study, a comprehensive bibliometric analysis was conducted utilizing two scientific literature visualization software packages: ScientoPy (version 2.1.3) and VOSviewer (version 1.6.17). To ensure a more comprehensive analysis, a dataset of 6,524 relevant publications on environmental behavior published in the Web of Science and Scopus spanning the past 50 years (1974–2024) was collected. The specific objectives are as follows: (1) To explore the spatial and temporal distribution of environmental behavior research, as well as the contributions of journals, disciplines, research institutions, and authors. (2) To reveal the thematic network and knowledge framework of environmental behavior research over the past 50 years. (3) To elucidate the development process and emerging trends of environmental behavior by integrating the outcomes of quantitative analysis and scientific mapping visualization, and to pinpoint the research frontiers. This will offer a reference for researchers in this domain to precisely understand the research directions and hot topics.

## 2 Methods

### 2.1 Data retrieval and processing

**Figure 1:**
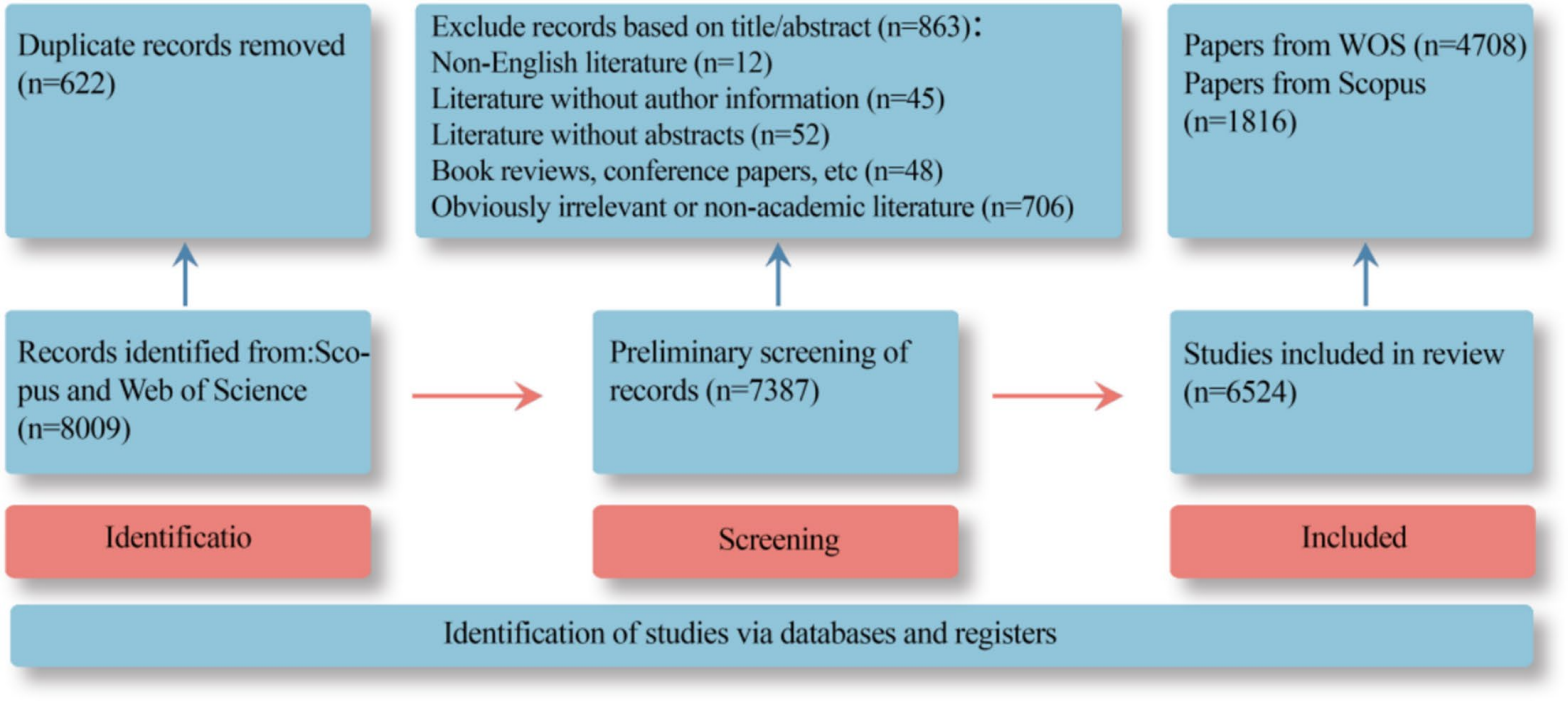
Flowchart illustrating the literature selection and database construction process.

This paper conducted literature retrieval using Web of Science and Scopus as data sources. To ensure data accuracy, the following search parameters were set in the Web of Science Core Collection (hereinafter referred to as WOS): (TS = (“environment behavior*”) OR ALL = (“environment engagement*”) OR TS = (“pro environment* behavior*”) OR TS = (“environment friendly behavior*”) OR TS = (“green behavior*”) OR TS = (“ecological behavior*”) OR TS = (“environment responsible behavior*”) OR TS = (“environment significant behavior*”) OR TS = (“environment sustainable behavior*”) AND ALL = (“environment* behavior*”)), with a time span from January 1, 1994 to December 31, 2024 (based on the indexing date), This resulted in 5,328 relevant documents. In the Scopus database, the search parameters were set as: (TITLE-ABS-KEY (“environment behavior*”) OR ALL (“environment engagement*”) OR ALL (“pro environment* behavior*”) OR ALL (“environment friendly behavior*”) OR ALL (“green behavior*”) OR ALL (“ecological behavior*”) OR ALL (“environment responsible behavior*”) OR ALL (“environment significant behavior*”) AND ALL (“environment behavior*”)), with a time range from 1974 to the present. This retrieved 2,681 relevant papers. By merging the literature from the WOS and Scopus databases, an initial total of 8,009 documents were obtained. After duplicate removal using EndNote: first, 14 duplicate documents in the WOS database were eliminated, leaving 5,314; then 608 duplicate documents in the Scopus database were removed, leaving 2,073. Ultimately, 7,387 documents were obtained. For the sake of the scientific nature of the data, further exclusion was carried out based on the title, abstract, etc. for non-English documents, documents without author information, documents without abstracts, and obviously irrelevant documents, totaling 863. Finally, 6,524 valid documents were retained, including 4,708 from the WOS database and 1,816 from the Scopus database. The literature screening process is detailed in Figure 1. This study conducted an in-depth analysis of the 6,524 screened documents.

### 2.2 Quantization performance analysis

In this study, we used ScientoPy (version 2.1.3) to calculate the number of articles published each year, revealing the growth of literature on environmental behavior from 1974 to 2024. At the same time, similar methods were employed to analyze the contributions of different countries, journals, disciplines, and authors to the field of environmental behavior research.

### 2.3 Scientific mapping analysis

This study employed keyword and term co-occurrence analysis to demonstrate the evolutionary trends of research hotspots within the field of environmental behavior and the interrelationships among its various sub-fields. Specifically, keyword co-occurrence analysis was used to identify the most prominent topics in environmental behavior research and their inherent connections. Term co-occurrence analysis, on the other hand, aimed to depict the focus and evolution of environmental behavior research by constructing a bibliometric map of terms.

#### 2.3.1 Keyword preprocessing and term extraction

In order to accurately identify research hotspots, we preprocessed the keywords of 6,524 articles and merged synonyms and related terms through standardized processing to reduce redundancy and clearly highlight the research hotspots. Using VOSviewer software, we selected keywords that appeared more than 25 times. As a result, 425 keywords related to “environmental behavior” were retained for further analysis.

#### 2.3.2 Co-occurrence analysis of keywords and terms

The occurrence frequency and average occurrence time of keywords were extracted by VOSviewer software, and the term co-occurrence analysis was carried out. In order to construct a bibliometric network based on term co-occurrence, and to reveal the relationship between keywords and research hotspots over time through network visualization technology. Among them, co-occurrence refers to the number of times that two terms appear together in the same paper, and the average occurrence time of the term refers to the average publication time of the literature containing the term (Ruiz-Rosero et al., 2019; Shahzad et al., 2025).

### 2.4 Trend analysis

#### 2.4.1 Average growth rate

In order to clarify the development trend of environmental behavior research from the perspective of countries, journals, disciplines and authors, the researchers used the equation to calculate the average growth rate of publication (AGR).


$$\mathrm{AGR}=\frac{\sum_{i=Y_{s}}^{Y_{e}}P_{i}-P_{i-1}}{(Y_{e}-Y_{s})+1}$$

In the AGR, AGR represents the average growth rate; Y_s_ and Y_e_ denote the beginning and ending years of the selected period, respectively; P_i_ represents the number of publications in year i. For an object, a rising trend attribute must have a higher publication growth rate, which can be reflected by AGR.

#### 2.4.2 Percentage of literature in recent years

Through the “percentage of literature in recent years” to study the hot journals, disciplines and keywords of environmental behavior related research, the formula is shown as:


$$\text{PDLY}=\frac{\sum_{i=Y_{s}}^{Y_{e}}P_{i}}{(Y_{e}-Y_{s}+1)\cdot \mathrm{TND}}\cdot 100\%$$

Among them, PDLY is the percentage of recent literature; Y_e_ = end of year; Y_s_ = the beginning of the year, Y_s_ = Y_e_ − (WindowWidth + 1); p_i_ = the number of publications in year i; TND = total number of literatures. The Window (year) is set to 5 during the calculation.

#### 2.4.3 Normalized cumulative frequency and trend factors

Trend analysis of specific keywords was conducted to reveal the temporal evolution of the research topic. The co-occurrence of keywords and terms was chosen for additional analysis, and the published literature from the past 6 years was divided into two groups according to the publication time (2019–2021 and 2022–2024). This was done to visualize the evolution of the selected keywords and quantify their potential for further development. According to Zhu et al. (2021), the normalized cumulative keyword frequency and trend factor are defined, with some modifications. Specifically, the normalized cumulative keyword frequency (NCF) is defined as the average number of occurrences of a keyword per 1,000 publications over a period of time (Zhu et al., 2021) The trend factor is calculated using the logarithm of the normalized cumulative keyword frequency ratios for 2019–2021 and 2022–2024. NCF quantifies the popularity of a topic in environmental behavior research. If a keyword has a lower NCF in 2019–2021 and a higher NCF in 2022–2024, the trend factor is positive, indicating an upward trend, and vice versa. In addition, the absolute value of the trend factor quantifies the magnitude of the upward or downward trend. The calculation formulas of NCF value and the trend factor are as follows:


$$F_{n(Y_{e}-Y_{s})}=\frac{\sum_{i=Y_{s}}^{Y_{e}}f_{i}}{\sum_{i=Y_{s}}^{Y_{e}}P_{i}}\times 1000$$

$$F_{\mathrm{nyr}}=\frac{f_{\mathrm{yr}}}{P_{\mathrm{yr}}}\times 1000$$

$$\text{trend factor}=\log(\frac{F_{n(2022-2024)}}{F_{n(2019-2021)}})$$

Where F_n_ (Y_e_ − Y_s_) denotes the NCF between Y_s_ and Y_e_; F_nyr_ denotes the Y_r_-year NCF; F_i_ represents the number of occurrences of i keywords.

## 3 Results

### 3.1 General overview of environmental behavior research field

#### 3.1.1 Analysis of the number of published papers on environmental behavior

**Figure 2:**
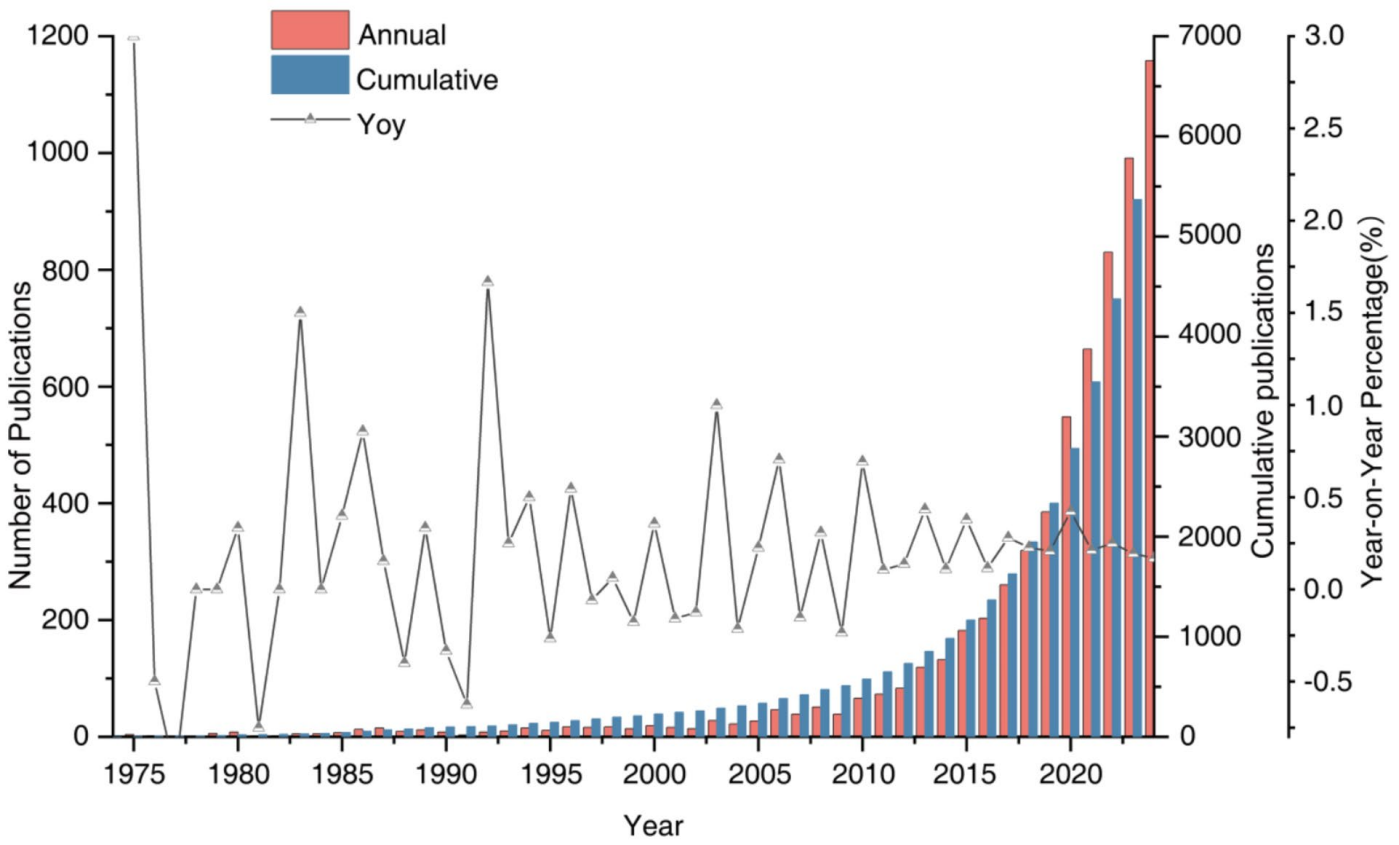
Evolution of environmental behavior-related publications in Web of Science and Scopus database.

The study found that the total number of publications related to environmental behavior in the two databases, WOS and Scopus, has demonstrated a rapid growth trend, increasing from 1 publication in 1974 to 1,158 in 2024 (Figure 2). Between 1974 and 2002, the number of publications in this field remained relatively low. This was primarily because environmental behavior had not yet gained widespread recognition and attention as a research topic. Moreover, the research foundation was underdeveloped, resulting in relatively slow growth of relevant literature. However, after the United Nations Sustainable Development Summit in 2002, research began to focus more on how to promote sustainable development by influencing human behavior. As the issue of climate change became increasingly urgent, research gradually shifted to investigating how policy tools could be used to influence public behavior. In 2003, the number of publications in environmental behavior research showed a distinct growth inflection point, with a growth rate of 1. From 2003 to 2012, this period represented a phase of rapid growth. During this period, the number of publications increased from 28 to 83. From 2003 to 2012, the number of publications rose from 28 to 83. This stage witnessed increasing academic interest, as the importance of environmental behavior became more widely recognized due to escalating environmental challenges. From 2013 to 2024, the number of publications in this field maintained a steady growth trend, indicating that environmental behavior research has become a prominent area of interest within the academic community. The fluctuations in publication numbers across different years also reflected the influence of factors such as the policy environment, societal trends, and advancements in academic research during those periods (Timmer et al., 2023; Maller, 2023). Overall, the number of publications in environmental behavior research exhibited a clear upward trend, which highlighted the growing importance and scholarly momentum in this field.

#### 3.1.2 Contributions of journals

**Figure 3:**
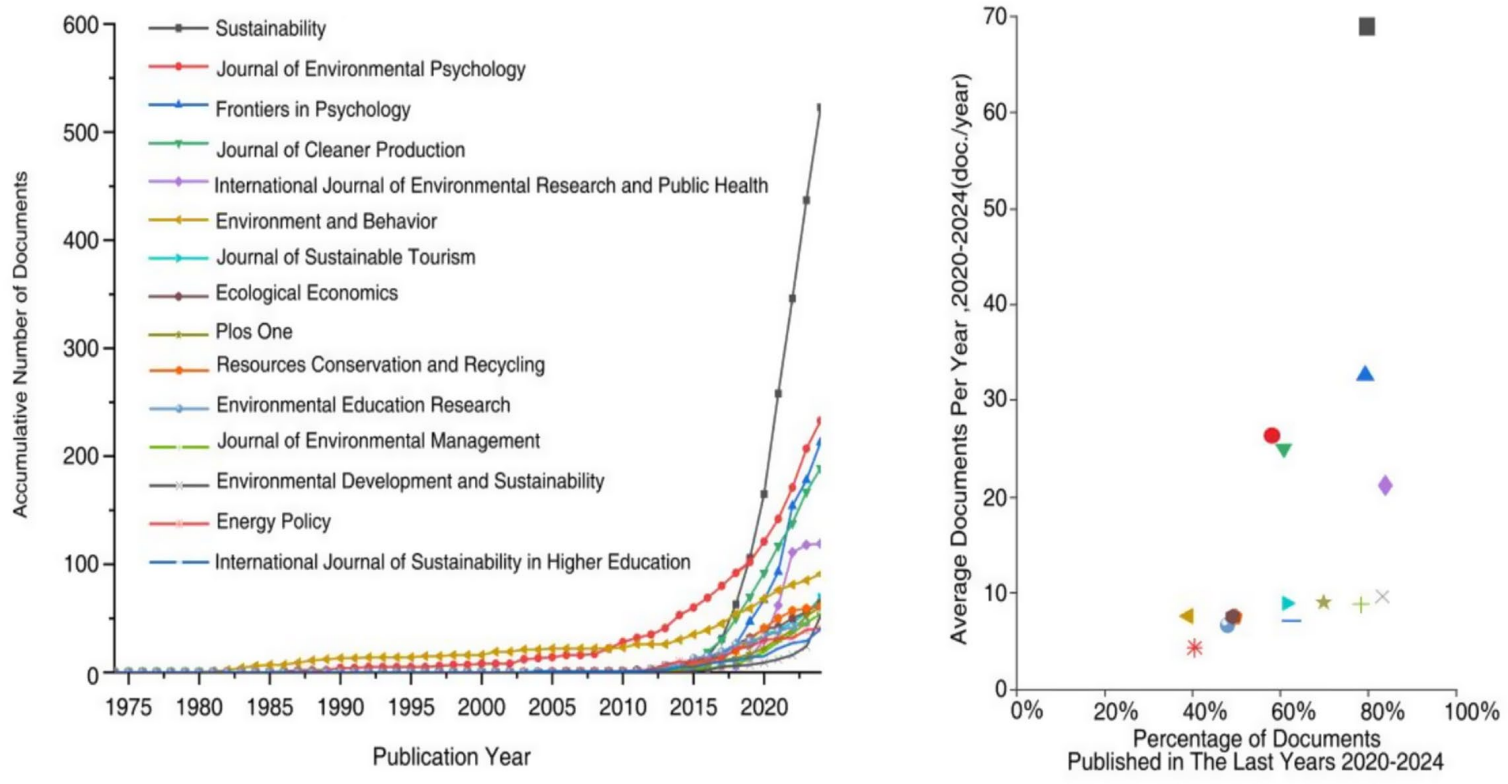
Evolution of published journals related to environmental behavior keywords in scientific network database.

By analyzing the number of papers published in journals related to environmental behavior (Figure 3), the results revealed that a total of 2,158 journals have published literature related to environmental behavior over the past 50 years. Among them, the top 15 journals have published a total of 1,864 articles, accounting for approximately one-third of all publications in this field. The top-ranked journal, *Sustainability*, has published 532 articles on environmental behavior. Following this, the *Journal of Environmental Psychology* published 233 articles, and *Frontiers in Psychology* published 213 articles, reflecting the significant role these journals play in the field of environmental behavior research. Further analysis revealed that journals such as the *International Journal of Environmental Research and Public Health* and *Environment Development and Sustainability* have maintained long-term focus on this area. Notably, over 80% of the literature in these journals has been published in the past 5 years, indicating increasing engagement of these journals with environmental behavior. The growing number of journals offer platforms for environmental behavior research, research, enabling scholars to share their latest findings and collaborate globally. These journals play a vital role in promoting scientific advancement and practical applications in the field of environmental behavior by fostering interdisciplinary collaboration, knowledge dissemination, and policy development (van Prooijen, 2019).

#### 3.1.3 Contributions of disciplines and institutions

**Figure 4:**
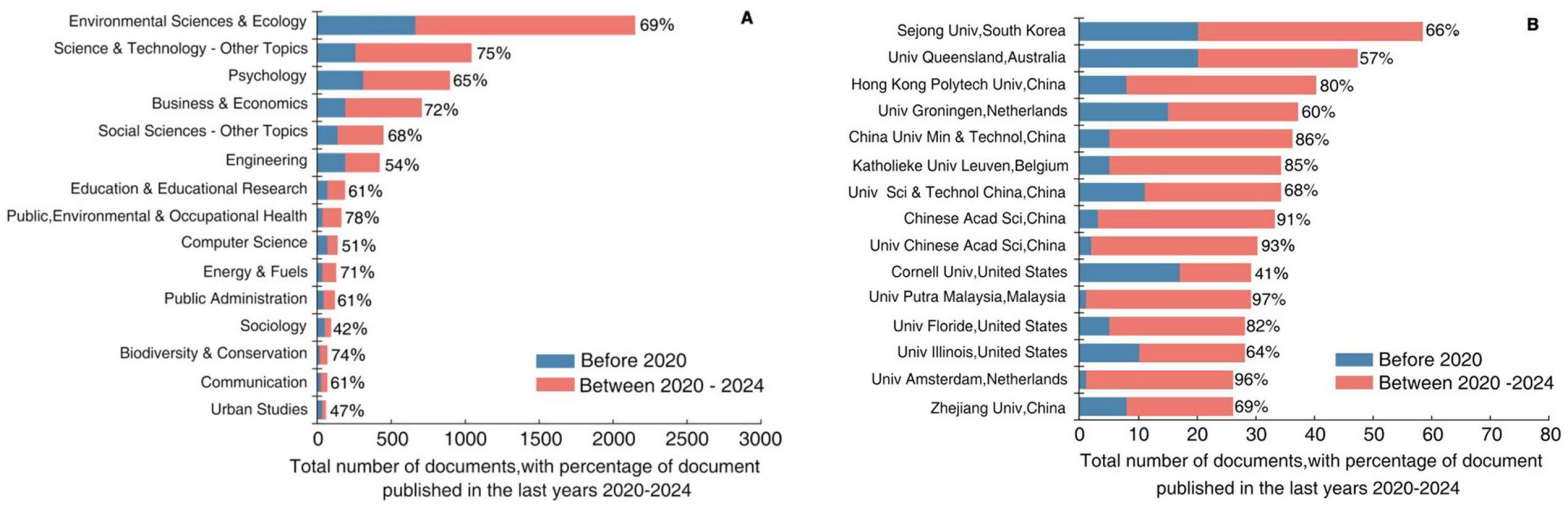
**(A)** Total number of documents and percentage of documents of the top 15 disciplines in environmental behavior -related research. **(B)** Total number of documents and percentage of documents of the top 15 research institution in environmental behavior -related research.

Research on environmental behavior spans 102 disciplines (Figure 4A). From a publication volume perspective, Environmental Sciences & Ecology was the most prominent field, with 2,142 related documents. This is followed by Science and Technology–Other Topics and Psychology, contributing 1,038 and 892 articles, respectively. These figures underscore environmental behavior as a shared focus across environmental science, ecology, science and technology, and psychology. Notably, approximately 70% of the literature in Business and Economics, Public, Environmental and Occupational Health, Energy and Fuels, and Biodiversity and Conservation has been published in the last 5 years, indicating a surged interest in these fields. From a disciplinary perspective, environmental behavior research exhibits increasing diversification. Over the past 5 years, additional disciplines, including Engineering, Education and Educational Research, Public Administration, Computer Science, and Biodiversity and Conservation, have increasingly engaged with this topic. This interdisciplinary approach not only enriches the theoretical framework of environmental behavior research but also expands its practical applications, facilitates knowledge exchange, and offers innovative approaches to complex environmental challenges (Hampton and Whitmarsh, 2023; Mosca et al., 2024).

A total of 4,076 institutions have conducted research on environmental behavior (Figure 4B). Among them, Sejong University in South Korea published the largest number of environmental behavior papers, leading with a total number of 58 publications This was followed by the University of Queensland in Australia, which published 47 papers; Hong Kong Polytechnic University in China, which published 40 papers; the University of Groningen in the Netherlands, which published 37 papers; and China University of Mining and Technology in China, which published 36 papers. Statistical analysis revealed that more than 85% of the environmental behavior research literature from institutions such as China University of Mining and Technology, the Chinese Academy of Sciences, the University of Chinese Academy of Sciences, Universiti Putra Malaysia, and University of Amsterdam has been published in the past 5 years. This demonstrated the significant research activity and continued investment of these institutions in the field of environmental behavior, highlighting their important contributions to the development of this field. The sustained contributions of these institutions would undoubtedly further advance development of environmental behavior research, driving innovation and breakthroughs in this field. From a national perspective, six of the top fifteen institutions with the largest total number of published documents were located in China. As a key academic research hub in Asia, China’s contribution to environmental behavior research stands out, which could be explained by the country’s strong emphasis on environmental protection and sustainable development. Additionally, research institutions in the United States and the Netherlands have demonstrated strong research capabilities, continuously promoting innovation in the field and making significant contributions to global environmental protection efforts. In summary, environmental behavior research has evolved into a cross-disciplinary, international field of study, attracting active participation and contributions from leading research institutions worldwide.

#### 3.1.4 Contribution of the author

The collaboration network of the most influential researchers and authors was further analyzed. Among them, Han from Sejong University in South Korea authored and co-authored the greatest number of papers, with a total of 49 publications. His research primarily focused on sustainable behavior models, the environmental behavior of cultivating customers, and the sustainability theory of green hotels and restaurants, supported by a broad and diverse collobration networks (Han, 2020, 2021; Han and Hyun, 2018; Han et al., 2017). Steg from the Netherlands followed closely with 30 co-authored papers. His work centered on developing comprehensive frameworks to encourage pro-environmental behavior, climate change psychology, and human factors related to sustainable energy transformation (Steg, 2016, 2023; Steg et al., 2021). Lange from Belgium co-authored 29 papers, focusing on the behavioral paradigms and influencing factors of pro-environmental behavior, with significant insights into the psychological mechanisms behind environmental behavior (Lange et al., 2014, 2024; Lange and Dewitte, 2021; Lange and Dewitte, 2020). In recent years, several emerging researchers have injected new vitality into the study of environmental behavior. Notably, the authors Li, Y., Zhang, Y., Brick, C., Liobikiene and Wang, J. have published more than 80% of their work in the past 5 years, highlighting their active and efficient contributions to environmental behavior research.

**Figure 5:**
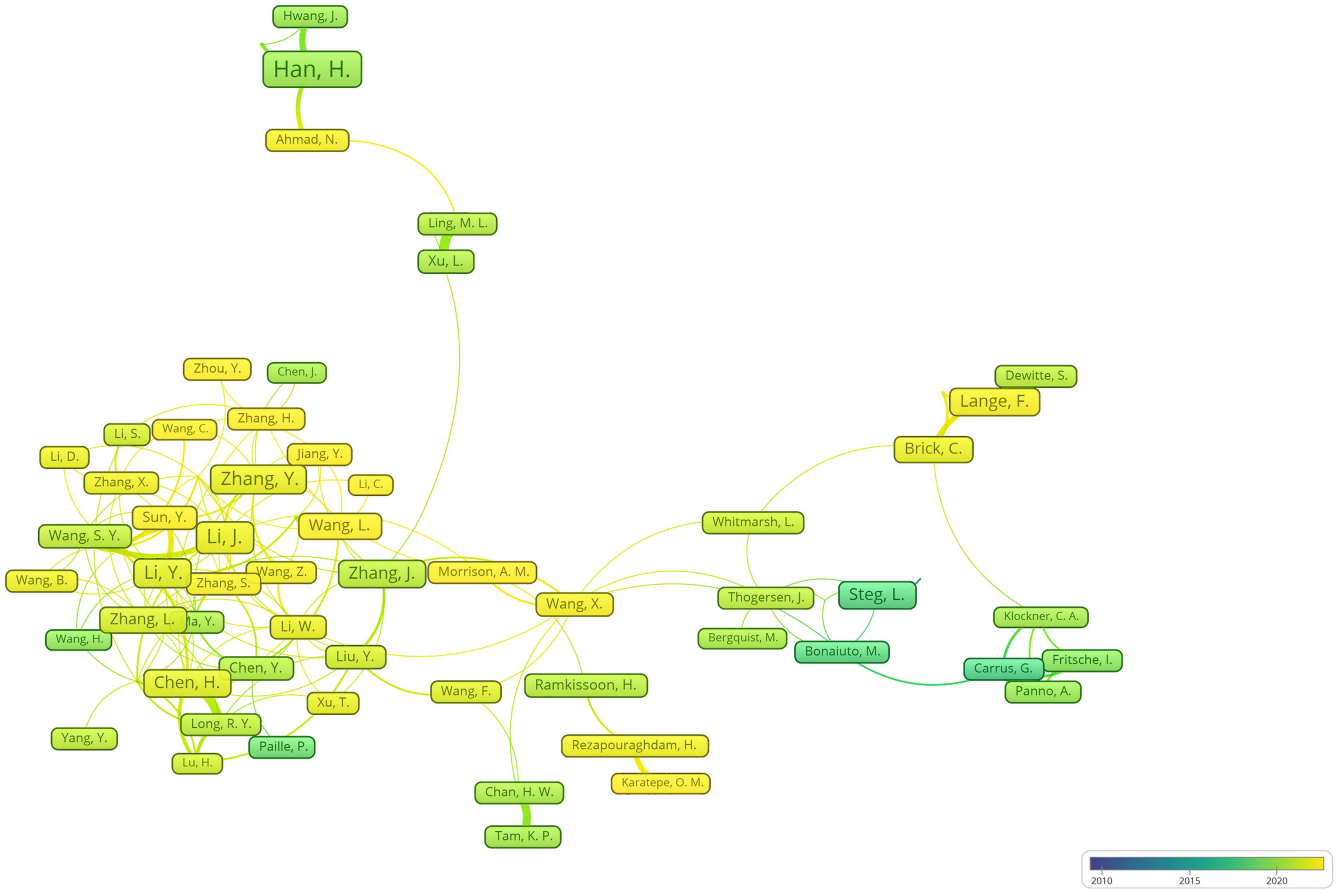
Author cooperation diagram.

With the application of VOSviewer to analyze the strength of cooperative links among authors in the field of environmental behavior, selection criteria were set for authors who had published 10 or more articles. The results showed that 87 authors out of a total of 17,124 met this criterion and were taken into consideration in the subsequent analysis (Figure 5). The author with the strongest collaborative connections was Han from South Korea, followed by Steg from the Netherlands and Lange from Belgium. These three authors exhibited the largest and most intense cooperative connections. The author collaboration network visualization reveals that a group of Chinese authors, including Li, J., Li, Y., Zhang, Y., Chen, H., and Wang, J., and Zhang, Y., has formed a close and strong network characterized by extensive and robust domestic collaboration, demonstrating significant research vitality. However, the scale and depth of their international collaboration networks remain limited. In contrast, the European author clusters centered around Steg from the Netherlands exhibits a strong collaboration network. A deeper analysis of these networks reveals that collaboration networks within developed countries (e.g., the Netherlands, Belgium, and the United Kingdom) are robust, while those with developing countries are relatively weak. To address this imbalance, developing countries should take proactive measures to expand their international collaboration opportunities in environmental behavior research. This includes establishing cross-regional collaborative bridges to facilitate emerging research clusters, particularly by strategically aligning researchers in developing countries and with those in the core European networks. Furthermore, creating knowledge-sharing platforms would enable countries to exchange experiences and technological advancements, enhancing international cooperation in environmental behavior projects to promote the sustainable development of global environmental behavior research.

### 3.2 Research process of environmental behavior: three stages

#### 3.2.1 Stages 1

**Figure 6:**
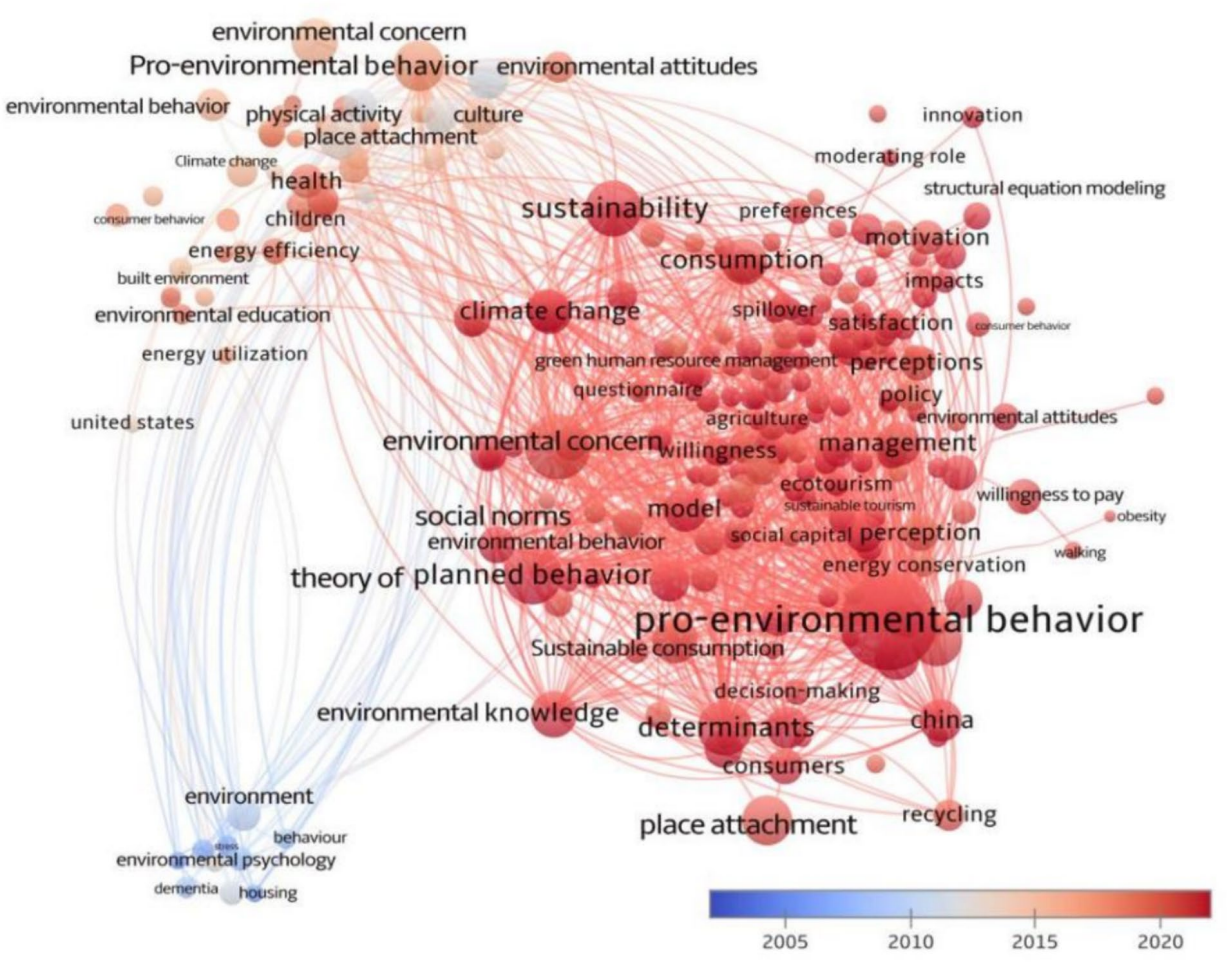
Keywords co-occurrence time graph.

Environmental behavior research emerged in the late 19th to early 20th century, driven by the deepening of the Industrial Revolution, accelerating urbanization, and the growing prominence of environmental issues. These factors prompted early scholars to explore the impacts of human activities on the natural environment. For instance, (1) First birth (Kruse and Graumann, 1987), (2) American transition (1930s to the end of World War II), (3) Architectural psychology (1950s to 1980s), and (4) Toward a “green” environmental psychology (late 80s to the present) (Pol, 2006, 2007, 2024). Through a systematic analysis of nearly 50 years of literature, as illustrated in the blue cluster of Figure 6, covering the period from 1974 to 2002, co-occurrence network analysis reveals frequent keywords such as “environment,” “behavior,” “housing,” and “environmental psychology.” These terms reflect the core focus of environmental behavior research during this period: the interaction between humans and their physical environment, with particular emphasis on the impact of built environments (e.g., housing, urban spaces, and workplaces) on behavior, well-being, and quality of life. In the 1970s, this field was often referred to as “architectural psychology,” concentrating on how spatial design, environmental stressors, and social contexts shape human experiences. For instance, Lévy-Leboyer, analyzed how environmental factors (such as urbanization, noise, and spatial design) affect an individual’s psychological state, behavior, and well-being, and explored how people perceive and adapt to environmental changes (Lévy-Leboyer, 1980); Bonnes emphasized the significance of understanding the relationship between humans and the environment from a social psychological perspective (Bonnes and Secchiaroli, 1995). As research deepened, the term “environmental psychology” was revisited in the 1980s. This return reflected an expansion in the scope of research, shifting from a focus solely on the built environment to a more comprehensive perspective that included studies from various age groups, cultural backgrounds, and social environments, highlighting the interaction between the environment, behavior, and social structure. For example, Kilbourne explored the role of the dominant social paradigm (DSP) in the formation of environmental attitudes and verified it through cross-national empirical research. Researchers employed interdisciplinary approaches, integrating knowledge from marketing, environmental psychology, sociology, and environmental science, to deeply analyze the complex mechanisms of environmental behavior. This has promoted a more comprehensive and in-depth understanding of the field and laid a solid foundation for subsequent research.

#### 3.2.2 Stages 2

The second stage, represented by the pink cluster in Figure 6 (2002–2012), is characterized by a keyword co-occurrence network that highlights core terms such as “pro-environmental behavior,” “environmental concern,” “environment,” “environmental attitudes,” “environmental behavior,” “environmental education,” “culture,” “physical activity,” “place attachment,” and “built environment.” Although these concepts were theoretically articulated in earlier stages, their significance markedly increased during this period. Research in this phase focused on promoting pro-environmental behaviors, with a strong emphasis on global environmental issues. It explored cultural differences across countries and addressed global environmental challenges, investigating how environmental attitudes, values, and norms drive sustainable behaviors.

In the second phase of environmental behavior research, “pro-environmental behavior” emerged as the most frequent keyword in environmental behavior research, underscoring its pivotal role in the field. Notably, research on pro-environmental behavior has been of critical importance since the 1990s (Scannell and Gifford, 2010; Kudryavtsev et al., 2012; Sánchez and Lafuente, 2010). The renewed prominence of “pro-environmental behavior” during this period reflects a significant shift from early theoretical explorations to a focus on specific behavioral practices. Its high frequency signifies a deepened scholarly inquiry into the mechanisms driving actual environmental actions, with particular emphasis on the psychological and social drivers of individual behavior, such as attitudes, values, and norms. For instance, Corral-Verdugo found a positive link between sustainable behaviors and personal well-being (Corral-Verdugo, 2002; Corral-Verdugo et al., 2011, 2013, 2020), while Steg provided a clear, comprehensive framework for understanding pro-environmental behavior by synthesizing scattered research across disciplines (Steg and Vlek, 2009). Fujii argued that environmental concern, frugal attitudes, and the perceived ease of implementation significantly influence pro-environmental behaviors (Fujii, 2006). These findings underscored the importance of pro-environmental actions for promoting environmental protection and sustainability (Nolan et al., 2008; Dietz et al., 2005; Ojala, 2012). The keyword “environment” remained central throughout this period, highlighting its fundamental role in behavior research (Turaga et al., 2010; Levine and Strube, 2012).

The emergence of keywords such as “environmental concern,” “environmental behavior,” and “environmental attitudes” highlights researchers’ growing focus on public’s awareness and perceptions of environmental issues, which are crucial for shaping effective conservation policies. For instance, Xiao, C. Y. examined gender differences in environmental behavior in China and demonstrated that the public’s environmental attitudes and attention directly affected their environmental behaviors (Xiao and Hong, 2010; Davis et al., 2009). Therefore, understanding and guiding the public to form correct environmental attitudes has become a critical direction in environmental behavior research (Davis et al., 2009; Wang and Li, 2022).

The frequent use of the keyword “environmental education” reflects researchers’ sustained focus on its role as a systematic tool for teaching environmental science and fostering public sensitivity, responsibility, and a connection to environmental issues. A sense of belonging often encourages positive environmental behaviors. Evidence shows that education programs incorporating community involvement and local knowledge, such as community gardens or local ecological monitoring, significantly enhance participants’ attachment to their surroundings and sense of duty, making them more likely to adopt daily sustainable practices. Scholars also explore how environmental education shapes social norms and personal moral obligations, indirectly influencing behavioral intentions and providing a foundation for designing effective intervention strategies to promote sustainable societies (Duerden and Witt, 2010; Matthies et al., 2012; Hashimoto-Martell et al., 2012). Simultaneously, the emergence of the “culture” keyword has broadened the research scope, drawing attention to the significant role that cultural background plays in environmental behavior. People from different cultural backgrounds may respond differently to the same environmental issues. For example, Ulijaszek studied the framework of population obesity and applied a cultural consensus model to explore the impact of the environment on population obesity (Ulijaszek, 2007). By incorporating cultural perspectives, researchers can gain a more comprehensive understanding of the diversity and complexity of environmental behavior, thus providing a stronger scientific foundation for developing effective environmental protection policies (Alves et al., 2005).

Keywords such as “place attachment,” “built environment,” and “physical activity” frequently appeared during this period. Such phenomenon reflected a sustained focus on the direct impact of spatial perception and the built environment on daily activity behaviors, particularly in the cross-field of urban planning and public health. During this stage, researchers not only focused on the environment itself but also began to delve into the interaction between people and the environment. For instance, “place attachment” made a recovery of its significance. This concept (then called “appropriation”) was first introduced at the IAPC conference in 1976 (Korosec-Serfaty, 1976), and its frequent appearance at this stage reflected people's emotional attachment and sense of belonging to specific locations or environments (Gosling and Williams, 2010; Raymond et al., 2011). The relationship between people and their surroundings is not merely functional but also serves as a carrier of emotions and identity, as Kudryavtsev argued that environmental education fostered emotional connections and value recognition toward specific places, thereby more effectively stimulating environmental awareness and behavior (Kudryavtsev et al., 2012). “Built environment” emphasizes how human-built environments shape and influence people’s behaviors and experiences (Smith, 2011; Kahn and Morris, 2009), For example, Pol’s focused on the impact of architectural design on human behavior and psychology (Pol, 2007; Pol, 2024). Meanwhile, “physical activity” highlights the influence of the environment on human health and activity levels. Researchers have started to explore how environmental design can promote healthier behaviors, such as increasing outdoor activity time and enhancing community environmental quality (Lee and Moudon, 2004; Cunningham and Michael, 2004). The frequent occurrence of these keywords indicates that environmental behavior research has entered a new developmental stage, offering a richer and more comprehensive perspective for future studies.

In summary, the research methods used to study environmental behavior in the second stage have become increasingly diverse and refined. These methods include experimental design, model simulations, long-term tracking studies, physiological measurement technologies, and other approaches to exploring both the internal mechanisms and external influencing factors of environmental behavior (Barbarossa and De Pelsmacker, 2016; Abd Rahman and Scaife, 2012). As research deepens, environmental behavior science has gradually integrated with other disciplines, such as sociology, psychology, and geography. This has resulted in an interdisciplinary research paradigm and laid a solid foundation for future studies.

#### 3.2.3 Stages 3

The third stage, represented by the red cluster in Figure 6, spans from 2013 to 2024, characterized by cross-disciplinary integration and pratical applications. Keywords include “pro-environmental behavior,” “sustainability,” “climate change,” “theory of planned behavior (TPB),” “place attachment,” “environmental concern,” “social norms,” “environmental education,” “environmental knowledge,” and “recycling” were first introduced in the 1970s and 1980s. However, in this stage, these keywords have acquired new meanings and greater research depth. With the intensification of global environmental challenges, the research focus has shifted toward translating theoretical frameworks into actionable intervention strategies. This period was characterized by multidisciplinary integration. Researches combined insights from environmental psychology, sociology, and behavioral science to investigate how policy formulation, educational initiatives, and social movements can enhance public environmental awareness to address urgent global issues such as climate change, biodiversity loss, and resource depletion.

“Pro-environmental behavior” has been the core keyword throughout the entire research process. During this period, research on pro-environmental behavior has witnessed explosive growth. Especially after 2012, the number of published papers has increased significantly each year, reaching a peak of over 300 per year in recent years, indicating that this field has become a popular research direction. Compared with the second stage, researchers have deepened their studies on pro-environmental behavior, and there have been obvious changes in the quantity of literature, as well as the breadth and depth of content. The research has gradually shifted from single-dimensional exploration to diversified comprehensive research, and the focus has shifted from “why” to “how.” Research is no longer satisfied with merely explaining behavior but has begun to explore effective behavioral intervention strategies. For instance, role models, social norms, “nudging” strategies in behavioral economics, and emotional appeals have all become popular research directions. For example, Truelove discussion the spillover effects of positive and negative environmental behaviors (Truelove et al., 2014). Moreover, Kalkbrenner and Roosen analyzed how community identity, social norms, trust, and environmental concern promote or limit citizens’ willingness to participate in community energy plans (Kalkbrenner and Roosen, 2016; Yuriev et al., 2020); and explored the perspective of the Theory of Planned Behavior on pro-environmental behavior. As research deepened, the focus of environmental behavior research has gradually expanded to different fields and levels. For instance, researchers have focused on the motivations for pro-environmental behavior at the individual level, such as the influence of values, attitudes, and beliefs on pro-environmental behavior (Venhoeven et al., 2016; Meyer, 2015; Kim and Seock, 2019). Some researchers have explored from the social level how policies, culture, and economic factors shape and regulate pro-environmental behavior (Larson et al., 2015; Barragan-Jason et al., 2023). These studies not only enrich the theoretical framework of pro-environmental behavior but also provide a scientific basis for environmental protection strategies in practice.

Keywords such as “sustainability” and “climate change” reflect the increasingly severe global environmental challenges and the growing attention from the academic community to issues related to sustainable development and climate change. “Sustainability,” as the second major focus at this stage, has prompted researchers to explore the pathways to sustainable development from various perspectives. For example, some researchers have focused on green consumption and sustainable lifestyles, analyzing the impact of changes in consumer behavior on environmental protection (Paço and Lavrador, 2017; Bimonte et al., 2022). Other scholars have concentrated on the role of businesses in sustainable development, discussing corporate social responsibility and environmental management strategies (Isensee et al., 2020; Islam et al., 2021; Martins et al., 2021). Investigating how nature relatedness connects well-being, environmental sustainability and pro-environmental behavior, research has found that it can promote sustainable lifestyles and relieve climate anxiety. “Climate change” is the third key keyword in this stage, and many researchers have engaged in in-depth discussions. They have studied how perceptions of climate change affect public environmental behavior. For instance, Bradley proposed strategies to promote pro-environmental behavior by enhancing green identity, response effectiveness, and psychological adaptation, which contribute to climate change mitigation. Researchers have also investigated how to further stimulate the public's enthusiasm for taking practical action to address climate change through education and policy incentives (Bradley et al., 2020; Wang et al., 2018; Zhang et al., 2020; Ogunbode et al., 2022). Additionally, researchers focused on the impact of climate change on natural ecosystems and socio-economic systems and proposed strategies for climate change adaptation and mitigation; they exploring how cross-cultural differences and age factors influence climate attitudes and behaviors, especially in the context of globalization and diversity (Panno et al., 2018; Wang et al., 2021). At the same time, keywords such as “environmental concern,” “environmental education,” and “environmental knowledge” have remained core themes in environmental behavior research, consistently shaping public attitudes and behavior toward environmental issues (Yang and Arhonditsis, 2022; Lee et al., 2014). Researchers have explored public concern about environmental issues, the effectiveness of environmental education, and the potential impact of environmental knowledge on behavioral change through extensive surveys (Wang et al., 2014; Liobikiene and Poskus, 2019). Enhancing public environmental awareness, strengthening environmental education, and promoting environmental knowledge can effectively encourage the adoption of more sustainable behaviors (Varela-Candamio et al., 2018). These findings not only deepen our understanding of environmental behavior but also provide a scientific foundation for developing effective environmental protection policies (Vicente-Molina et al., 2018; Naz et al., 2023).

In addition, keywords such as “theory of planned behavior,” “place attachment,” “social norms,” and “recycling” received increasing attention from researchers. These keywords indicate that the research in environmental behavior has evolved toward a more detailed and in-depth approach. Using the theory of planned behavior (TPB), researchers have explored how personal attitudes, social norms, and perceived behavioral control influence public environmental behavior (Han, 2015; De Leeuw et al., 2015). The study of “place attachment” has become more interdisciplinary. The focus shifting to integrating local attachment into a broader social-environmental framework to address global challenges such as climate change and urban sustainability (Ramkissoon et al., 2013; Gifford, 2014; Hosany et al., 2017). The keyword “social norms” reflects how social norms shape public perceptions and expectations of environmental behavior, thereby encouraging more people to take environmental action (Thomas and Sharp, 2013). The keyword “recycling” addresses the factors that influence public recycling behavior and proposes effective strategies to promote recycling. These research findings provide a scientific foundation for formulating more effective environmental protection policies (Echegaray and Hansstein, 2017; Winterich et al., 2019; Mi et al., 2019).

At this stage, interdisciplinary collaboration in environmental behavior research has become the norm. Environmental behaviorists, sociologists, psychologists, geographers, and educators are working together to explore the multi-dimensional influencing factors and effective intervention strategies for environmental behavior. This interdisciplinary research model has significantly enriched the theoretical framework of environmental behavior and driven innovation in practical applications. In terms of research methods, the use of mobile devices, sensor technology, Geographic Information Systems (GIS), Virtual Reality (VR), and Augmented Reality (AR) has enhanced the depth and accuracy of research. These tools also provide strong support for the development of targeted environmental behavior intervention strategies. Furthermore, research at this stage emphasizes the importance of public participation, encouraging the public to actively engage in both the research and practical aspects of environmental behavior. Therefore, this approach accelerates the transition toward a greener society through interdisciplinary and methodological innovation.

### 3.3 Trend analysis of environmental behavior

#### 3.3.1 Trend analysis of countries studying environmental behavior

**Figure 7:**
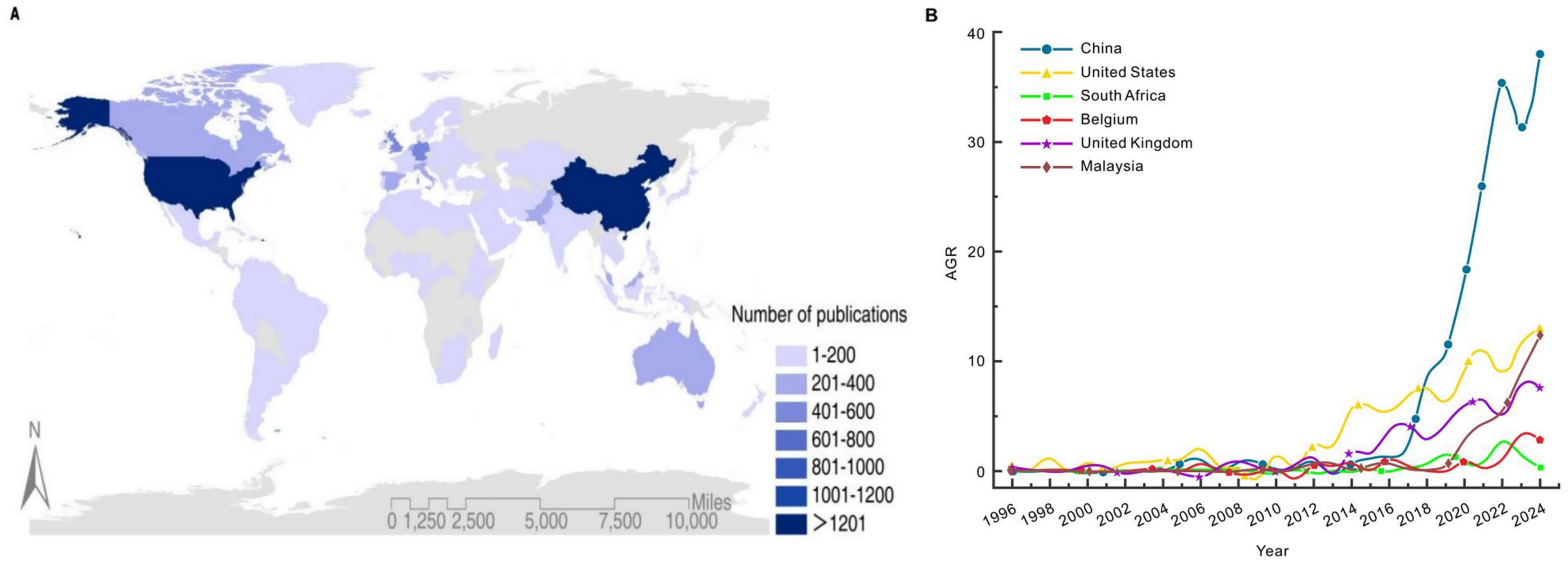
**(A)** Geographical distribution of studies on environmental behavior by country. **(B)** Trends in the growth rate of environmental behavior research in China, the United States, the United Kingdom, Malaysia, Belgium and South Africa.

As shown in Figure 7A, an analysis of the geographical distribution of environmental behavior studies based on the corresponding authors’ countries or their countries of origin reveals that the authors of these papers come from 144 countries. Among them, China (1486), the United States (1325), and the United Kingdom (546) are the top three countries in terms of environmental behavior research output. This might be related to the national conditions, such as the rapid economic development in China leading to serious environmental problems, which has driven research to address pollution and climate change. The United States has a long tradition of research in the fields of environment and psychology, with abundant research funds, top universities, and a well-established publishing system ensuring its leading position in scientific research. The United Kingdom, as the birthplace of the Industrial Revolution, has a deep historical understanding of the environment and a strong academic foundation and global publishing influence, enabling its achievements to remain ahead. Additionally, Germany, Australia, Canada, Italy, Malaysia, Spain, and the Netherlands rank among the top 10 countries with the highest number of publications in this field. These countries not only produce a large volume of research in environmental behavior but also own more established research teams and have abundant scientific resources in this domain. With the acceleration of globalization, environmental behavior research increasingly shows characteristics of international collaboration. Researchers from different countries have jointly promoted the development of environmental behavior research by sharing data, exchanging experiences, and conducting joint research projects. Such international collaboration not only broadens the research scope and improves research quality, but also fosters coordination and cooperation among countries in the formulation and implementation of environmental policies.

As shown in Figure 7B, we use the Average Growth Rate (AGR) method to further calculate the AGR based on the number of papers published by different countries. This enables us to obtain the AGR values for environmental behavior literature published by countries over the past 30 years, divided into 5-year intervals. Six representative countries—China, the United States, the United Kingdom, Malaysia, Belgium, and South Africa—were selected for analysis. As the country with the largest number of publications, China’s AGR has shown explosive growth in recent years. Based on the current data, China is expected to continue drive innovation in environmental behavior research in the future, contributing more scientific insights and practical experience to global research in this field. The United States was pioneered in researching environmental behavior, and its AGR curve has risen steadily in waves. While research interest has increased gradually, the overall trend remains stable, with a potential peak expected in the coming years. The publication trends in Belgium and the United Kingdom are similar, with both countries experiencing a decline after their respective peaks. The publication trends of Belgium and the United Kingdom are similar, both experiencing a peak and then a decline. Perhaps due to Brussels, the headquarters of the European Union, is a strong promoter of international research, it has gathered most of the research produced by different European countries. This research has entered the period of practical optimization, resulting in a marginal reduction in theoretical research output. Malaysia’s publications have steadily increased in recent years, and its AGR curve shows a “step-by-step leap forward” pattern, which is closely related to government-driven initiatives and the transition toward a green economy. As a developing country, South Africa has a smaller number of publications on environmental behavior and a lower AGR value. Currently, there is no clear trend, and the AGR has shown a decline in recent years, possibly due to limited research resources and insufficient government investment in environmental governance.

#### 3.3.2 Evolution analysis of environmental behavior keywords

**Figure 8:**
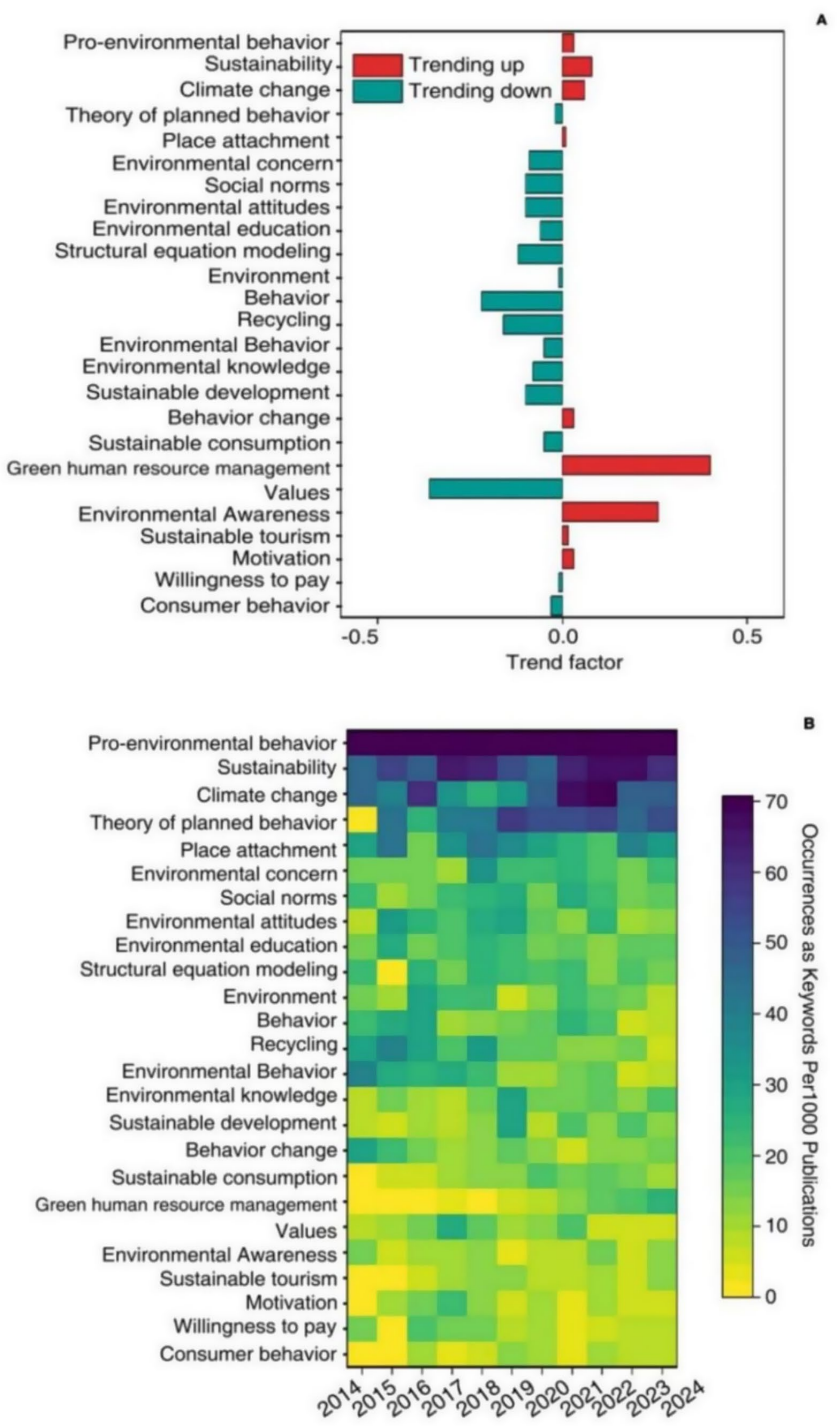
Evolution trend of the top 25 hot spots of environmental behavior in terms of the trend factor **(A)** Narmalized cumuhive frequency **(B)** Keywords of environmental behavior ranked in descending order of frequency of occurrence.

A systematic review of emerging research hotspots and academic frontiers in the field of environmental behavior not only provides a comprehensive overview of the discipline’s evolution, but also precisely identifies key topics, establishes a multidimensional theoretical framework, and offers an innovative action guide to support national strategies for environmental protection and sustainable development. To ensure the scientific rigor and forward-looking nature of the research conclusions, we employed a trend factor recognition algorithm and normalized cumulative frequency analysis to dynamically track and quantitatively assess keywords from a vast corpus of environmental behavior literature. We evaluated the trend evolution of environmental behavior research hotspots by analyzing the total number of publications, keyword frequency, and their temporal distribution (Figure 8A). Using normalized cumulative keyword frequency, we quantitatively traced the developmental trajectory of environmental behavior topics, based on keyword frequencies across every 1,000 publications from 2014 to 2024 (Figure 8B). The trend factors for “Pro-environmental behavior,” “sustainability,” “climate change,” “place attachment,” “environmental awareness,” “sustainable tourism,” “motivation,” “green human resource management,” and “behavior change” were positive, indicating significant growth in these areas in recent years. These trends reflect emerging hotspots and frontier directions in environmental behavior research. Among these fields, the growth trend of “green human resource management” (GHRM) is particularly notable, with a trend factor of 0.4. The NCF of GHRM surged from 3.85 in 2017 to 25.04 in 2024, an increase of about 5.5 times, highlighting GHRM as an emerging and rapidly developing subfield in environmental behavior research. This indicates that GHRM has attracted substantial attention and in-depth discussion and may lead to more innovations and breakthroughs in the field, offering new perspectives and methodologies for environmental behavior research (Darvishmotevali and Altinay, 2022; Nisar et al., 2021). Following this, “environmental awareness” has a trend factor of 0.26, ranking second compared to GHRM, and its NCF value also shows an upward trend. This reflects the continued focus on environmental awareness research. As environmental issues become increasingly severe, improving public awareness is crucial for promoting environmental protection (Martinho et al., 2015; Afsar et al., 2016; Fu et al., 2020; Barbaro and Pickett, 2016). The positive trend of this keyword suggests that future environmental behavior research will place more emphasis on practical effects and societal impacts, providing strong support for environmental protection. The positive trend factors for the keywords “sustainability” (0.08) and “climate change” (0.06) further indicate the ongoing global attention to these issues and society’s increasing awareness of ecological crises. From 2019 to 2024, “Pro-environmental behavior (PEB)” had the highest NCF value among the top 25 keywords, with an average of 267.96 and a positive trend factor of 0.03, demonstrating its enduring significance and influence in environmental behavior research (Barbaro and Pickett, 2016; Ho et al., 2015). The NCF values for keywords such as “behavior change,” “place attachment,” and “sustainable tourism” showed both upward trends and positive trend factors, highlighting the growing diversity and complexity of environmental behavior research (Naito et al., 2022; Kim and Lee, 2022; D'Arco et al., 2023). Interestingly, while the number of publications and NCF values for “theory of planned behavior” (TPB) are high, its trend factor is negative (−0.02). This small absolute value suggests not a decline in relevance but rather a maturation of the theory — with ongoing research entering phases of refinement rather than expansion. Its core position in the field remains stable, and its influence continues to be significant in environmental behavior research. On the other hand, keywords like “values” and “attitudes” have higher negative trend factors, indicating declining scholarly interest. The decreasing appeal of these abstract concepts may be attributed to a shift in focus toward more practical applications and specific solutions, rather than theoretical discussions. The high negative values for these trend factors reflect the evolving needs and expectations of both academic and practical domains in environmental behavior research.

## 4 Conclusion

This study employs co-occurrence analyses of journals, institutions, and authors to delineate the core journal clusters (e.g., *Journal of Environmental Psychology*, *Frontiers in Psychology*, etc.), leading research institutions (top-tier universities in Europe and North America, alongside emerging Asian institutions such as Sejong University in South Korea and Hong Kong Polytechnic University in China,etc), and collaborative networks among key researchers. These analyses reveal a global cooperative pattern characterized by interdisciplinary and inter-institutional synergy. Through burst detection and thematic evolution analyses, the study systematically traces the trajectory of environmental behavior research. The field evolved from sporadic studies in the late 19th to early 20th centuries to a focus on physical environments such as “housing” in the 1970s–1990s, emphasizing how individuals perceive and understand their environments. In the early 21st century, research shifted toward broader environmental issues, including “environmental education” “place attachment” and “culture” with an emphasis on the types of environmental behaviors individuals adopt. Over the past decade, the focus has transitioned to macro-level socio-ecological issues such as “sustainability” “climate change” and the “theory of planned behavior” alongside environmental well-being. It centers on how to effectively leverage policy interventions to change environmental behaviors. Using trend factor and normalized cumulative frequency analyses, the study identifies thematic shifts and frontier directions. The keywords “green human resource management” and “environmental awareness” emerge as the fastest-growing hotspots, reflecting the critical role of policy in global green transitions. These hotspots provide practical pathways for translating environmental awareness into sustainable behaviors through policy design and offer theoretical support for organizations to promote environmental responsibility via green human resource management. Meanwhile, enduring core themes such as “pro-environmental behavior” “sustainability” and “climate change” maintain their prominence. The coexistence of emerging hotspots and stable core themes indicates that environmental behavior research sustains its foundational issues while expanding into new interdisciplinary domains. This study constructs a knowledge map and elucidates the thematic evolution logic of environmental behavior research, providing systematic empirical support for understanding the field’s developmental trajectory. Furthermore it offers targeted practical guidance for policymakers, business leaders, and social organizations to drive environmental behavior transformations through its analysis of hotspots and core issues.

In the future, within the framework of interdisciplinary integration, environmental behavior research will increasingly focus on areas such as “green human resource management” “climate change” “sustainability” and “culture.” This involves integrating frameworks like Environmental, Social, and Governance (ESG) systems and dual-carbon goals to drive the transformation of sustainable corporate behaviors. These efforts will synergize with core themes such as “climate change” and “pro-environmental behavior” providing robust empirical evidence for policy design and fostering cross-disciplinary innovation. In the meantime, transnational cooperation will play a pivotal role, enabling countries to address the global climate crisis collaboratively through shared behavioral data and intervention strategies. Such cooperation will strengthen natural resource conservation, facilitate the realization of the Sustainable Development Goals (SDGs), and offer cross-cultural empirical support for policy formulation, ultimately accelerating the transition toward sustainable development.

## Data Availability

The original contributions presented in the study are included in the article/supplementary material, further inquiries can be directed to the corresponding author.
